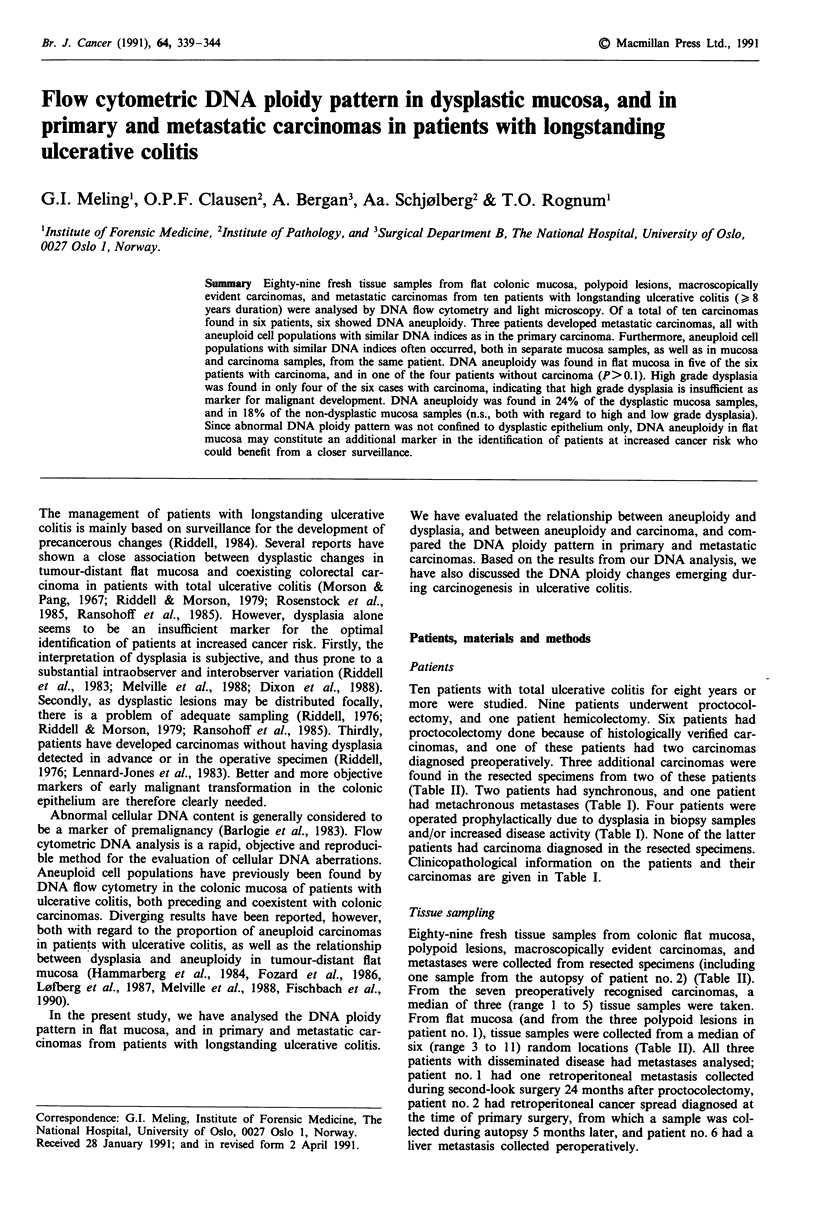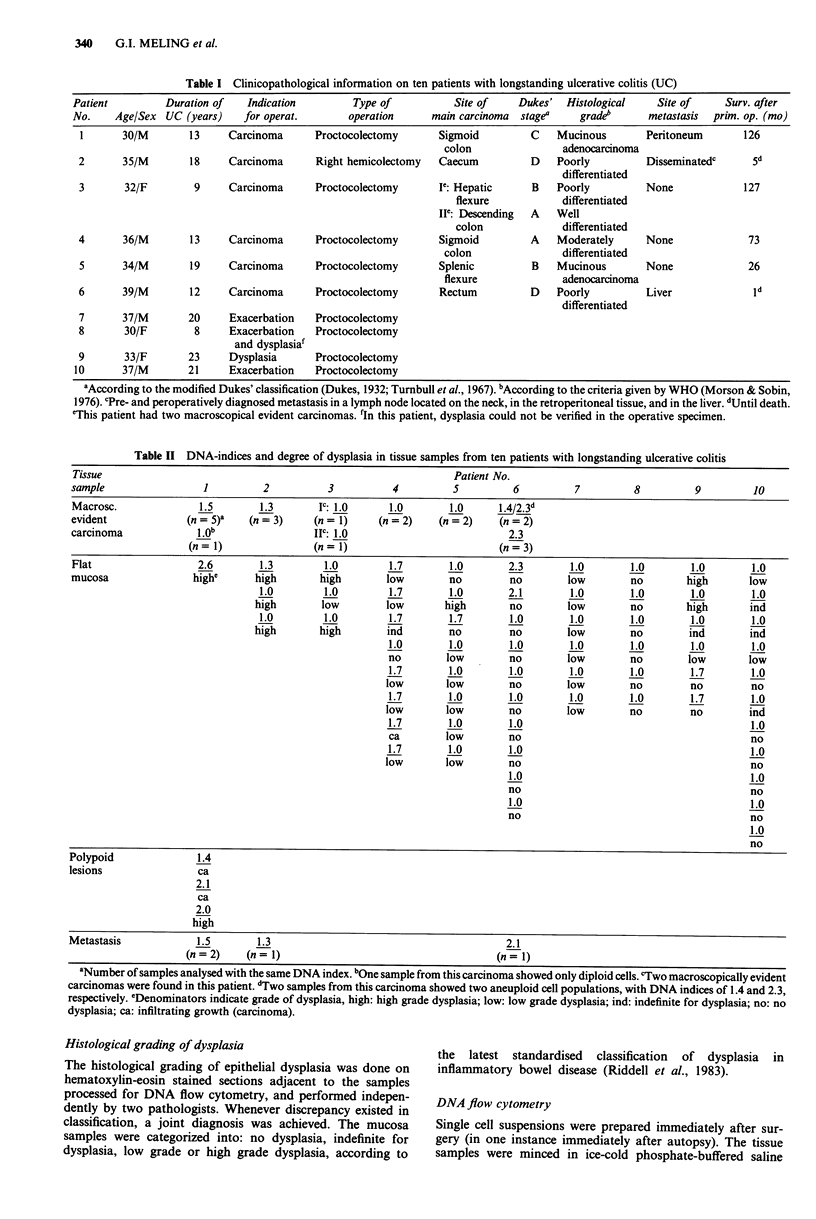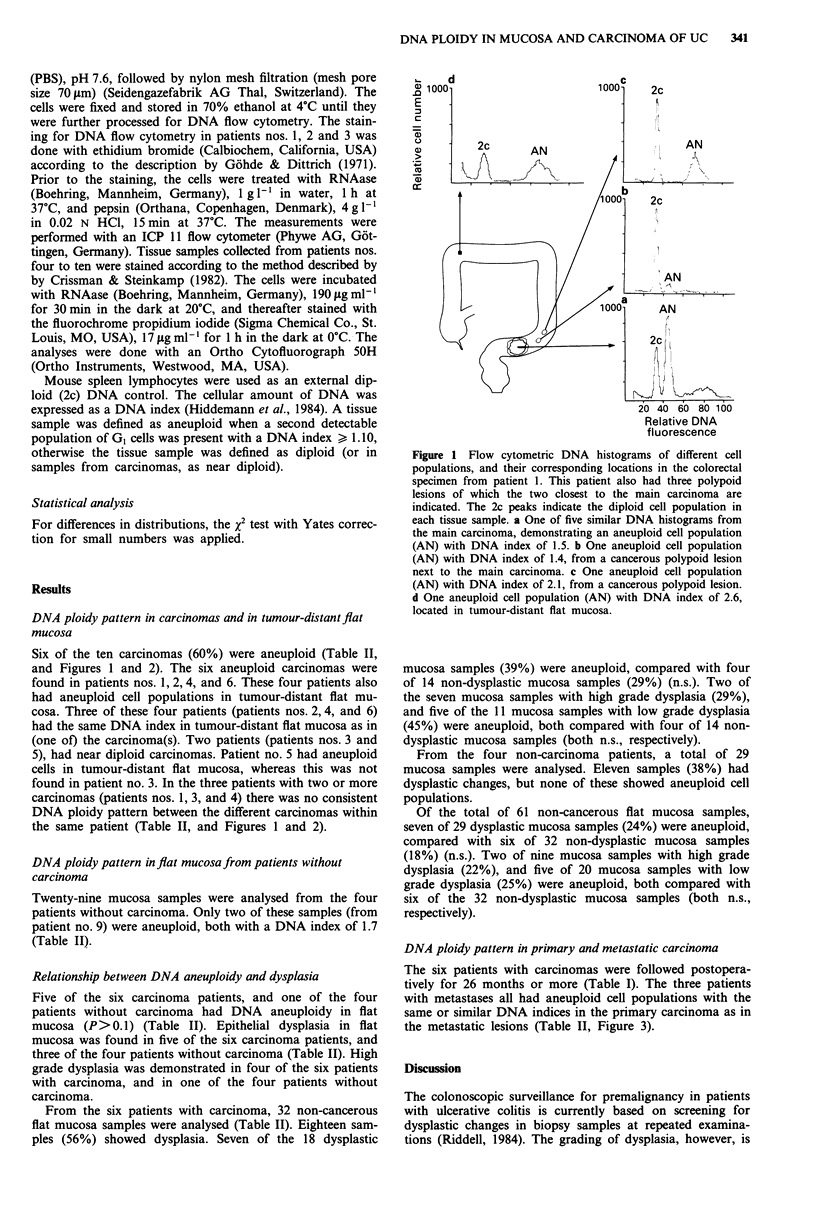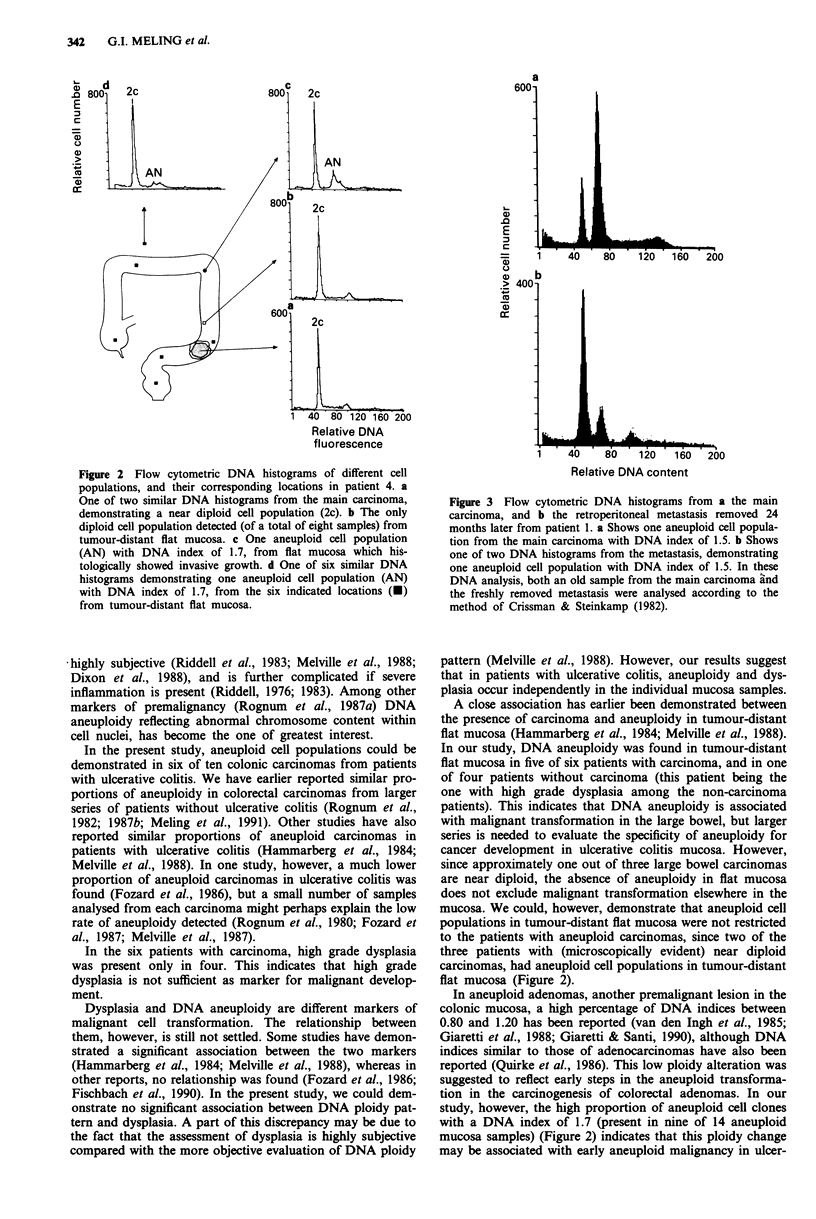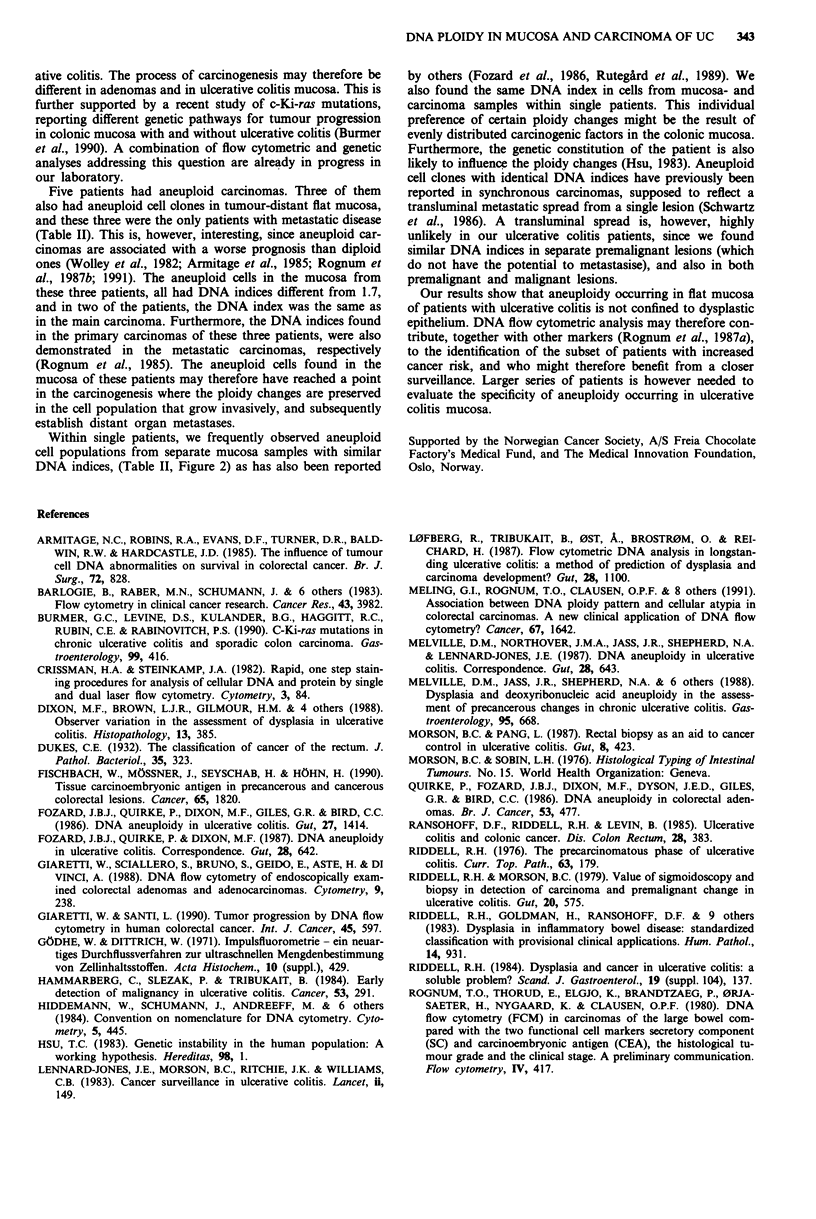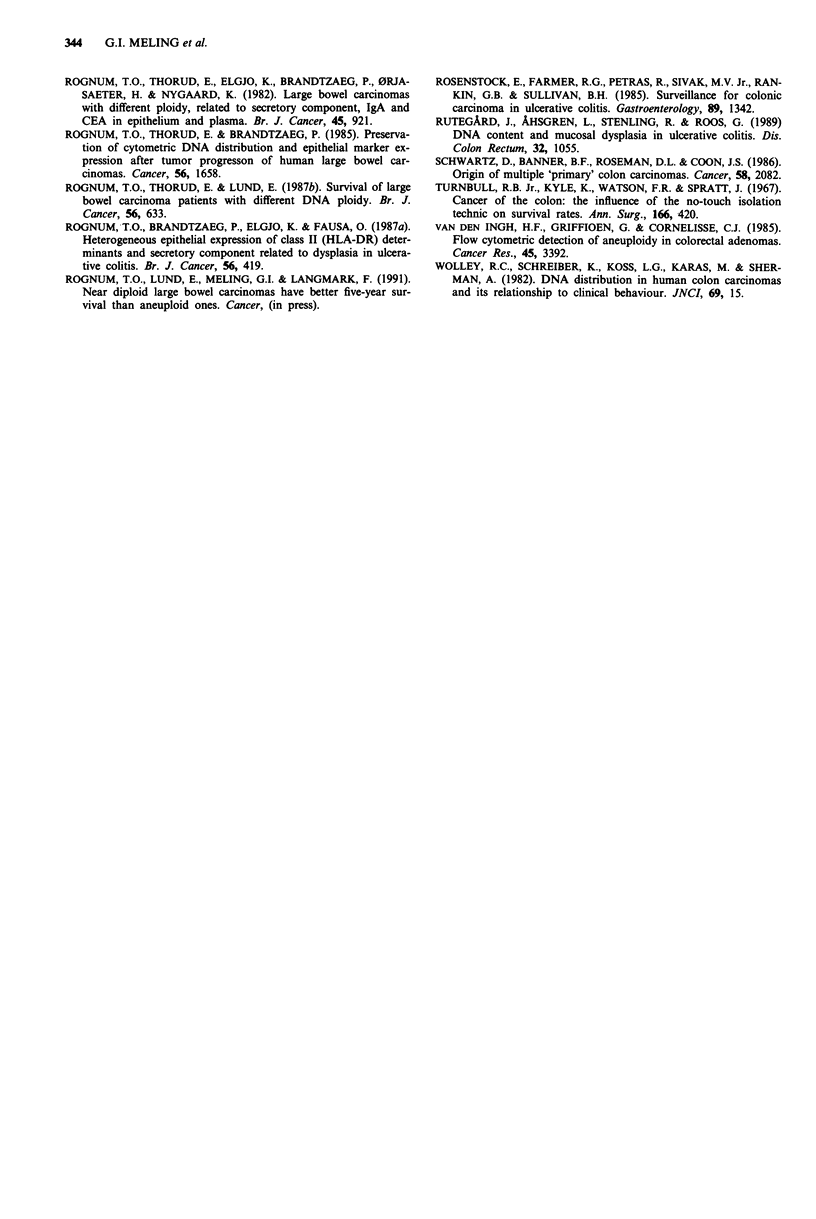# Flow cytometric DNA ploidy pattern in dysplastic mucosa, and in primary and metastatic carcinomas in patients with longstanding ulcerative colitis.

**DOI:** 10.1038/bjc.1991.302

**Published:** 1991-08

**Authors:** G. I. Meling, O. P. Clausen, A. Bergan, A. Schjølberg, T. O. Rognum

**Affiliations:** Institute of Forensic Medicine, National Hospital, University of Oslo, Norway.

## Abstract

Eighty-nine fresh tissue samples from flat colonic mucosa, polypoid lesions, macroscopically evident carcinomas, and metastatic carcinomas from ten patients with longstanding ulcerative colitis (greater than or equal to 8 years duration) were analysed by DNA flow cytometry and light microscopy. Of a total of ten carcinomas found in six patients, six showed DNA aneuploidy. Three patients developed metastatic carcinomas, all with aneuploid cell populations with similar DNA indices as in the primary carcinoma. Furthermore, aneuploid cell populations with similar DNA indices often occurred, both in separate mucosa samples, as well as in mucosa and carcinoma samples, from the same patient. DNA aneuploidy was found in flat mucosa in five of the six patients with carcinoma, and in one of the four patients without carcinoma (P greater than 0.1). High grade dysplasia was found in only four of the six cases with carcinoma, indicating that high grade dysplasia is insufficient as marker for malignant development. DNA aneuploidy was found in 24% of the dysplastic mucosa samples, and in 18% of the non-dysplastic mucosa samples (n.s., both with regard to high and low grade dysplasia). Since abnormal DNA ploidy pattern was not confined to dysplastic epithelium only, DNA aneuploidy in flat mucosa may constitute an additional marker in the identification of patients at increased cancer risk who could benefit from a closer surveillance.


*Br. J. Cancer* (1991), **64**, 339–344 © Macmillan Press Ltd., 1991

### Flow cytometric DNA ploidy pattern in dysplastic mucosa, and in primary and metastatic carcinomas in patients with longstanding ulcerative colitis

G.I. Meling[1], O.P.F. Clausen[2], A. Bergan[3], Aa. Schjølberg[2] & T.O. Rognum[1]

[1]*Institute of Forensic Medicine,* [2]*Institute of Pathology, and* [3]*Surgical Department B, The National Hospital, University of Oslo, 0027 Oslo 1, Norway.*

**Summary** Eighty-nine fresh tissue samples from flat colonic mucosa, polypoid lesions, macroscopically evident carcinomas, and metastatic carcinomas from ten patients with longstanding ulcerative colitis ($\geqslant 8$ years duration) were analysed by DNA flow cytometry and light microscopy. Of a total of ten carcinomas found in six patients, six showed DNA aneuploidy. Three patients developed metastatic carcinomas, all with aneuploid cell populations with similar DNA indices as in the primary carcinoma. Furthermore, aneuploid cell populations with similar DNA indices often occurred, both in separate mucosa samples, as well as in mucosa and carcinoma samples, from the same patient. DNA aneuploidy was found in flat mucosa in five of the six patients with carcinoma, and in one of the four patients without carcinoma ($P > 0.1$). High grade dysplasia was found in only four of the six cases with carcinoma, indicating that high grade dysplasia is insufficient as marker for malignant development. DNA aneuploidy was found in 24% of the dysplastic mucosa samples, and in 18% of the non-dysplastic mucosa samples (n.s., both with regard to high and low grade dysplasia). Since abnormal DNA ploidy pattern was not confined to dysplastic epithelium only, DNA aneuploidy in flat mucosa may constitute an additional marker in the identification of patients at increased cancer risk who could benefit from a closer surveillance.

The management of patients with longstanding ulcerative colitis is mainly based on surveillance for the development of precancerous changes (Riddell, 1984). Several reports have shown a close association between dysplastic changes in tumour-distant flat mucosa and coexisting colorectal carcinoma in patients with total ulcerative colitis (Morson & Pang, 1967; Riddell & Morson, 1979; Rosenstock *et al.*, 1985, Ransohoff *et al.*, 1985). However, dysplasia alone seems to be an insufficient marker for the optimal identification of patients at increased cancer risk. Firstly, the interpretation of dysplasia is subjective, and thus prone to a substantial intraobserver and interobserver variation (Riddell *et al.*, 1983; Melville *et al.*, 1988; Dixon *et al.*, 1988). Secondly, as dysplastic lesions may be distributed focally, there is a problem of adequate sampling (Riddell, 1976; Riddell & Morson, 1979; Ransohoff *et al.*, 1985). Thirdly, patients have developed carcinomas without having dysplasia detected in advance or in the operative specimen (Riddell, 1976; Lennard-Jones *et al.*, 1983). Better and more objective markers of early malignant transformation in the colonic epithelium are therefore clearly needed.

Abnormal cellular DNA content is generally considered to be a marker of premalignancy (Barlogie *et al.*, 1983). Flow cytometric DNA analysis is a rapid, objective and reproducible method for the evaluation of cellular DNA aberrations. Aneuploid cell populations have previously been found by DNA flow cytometry in the colonic mucosa of patients with ulcerative colitis, both preceding and coexistent with colonic carcinomas. Diverging results have been reported, however, both with regard to the proportion of aneuploid carcinomas in patients with ulcerative colitis, as well as the relationship between dysplasia and aneuploidy in tumour-distant flat mucosa (Hammarberg *et al.*, 1984, Fozard *et al.*, 1986, Løfberg *et al.*, 1987, Melville *et al.*, 1988, Fischbach *et al.*, 1990).

In the present study, we have analysed the DNA ploidy pattern in flat mucosa, and in primary and metastatic carcinomas from patients with longstanding ulcerative colitis. We have evaluated the relationship between aneuploidy and dysplasia, and between aneuploidy and carcinoma, and compared the DNA ploidy pattern in primary and metastatic carcinomas. Based on the results from our DNA analysis, we have also discussed the DNA ploidy changes emerging during carcinogenesis in ulcerative colitis.

Correspondence: G.I. Meling, Institute of Forensic Medicine, The National Hospital, University of Oslo, 0027 Oslo 1, Norway.
Received 28 January 1991; and in revised form 2 April 1991.

### Patients, materials and methods

### *Patients*

Ten patients with total ulcerative colitis for eight years or more were studied. Nine patients underwent proctocolectomy, and one patient hemicolectomy. Six patients had proctocolectomy done because of histologically verified carcinomas, and one of these patients had two carcinomas diagnosed preoperatively. Three additional carcinomas were found in the resected specimens from two of these patients (Table II). Two patients had synchronous, and one patient had metachronous metastases (Table I). Four patients were operated prophylactically due to dysplasia in biopsy samples and/or increased disease activity (Table I). None of the latter patients had carcinoma diagnosed in the resected specimens. Clinicopathological information on the patients and their carcinomas are given in Table I.

### *Tissue sampling*

Eighty-nine fresh tissue samples from colonic flat mucosa, polypoid lesions, macroscopically evident carcinomas, and metastases were collected from resected specimens (including one sample from the autopsy of patient no. 2) (Table II). From the seven preoperatively recognised carcinomas, a median of three (range 1 to 5) tissue samples were taken. From flat mucosa (and from the three polypoid lesions in patient no. 1), tissue samples were collected from a median of six (range 3 to 11) random locations (Table II). All three patients with disseminated disease had metastases analysed; patient no. 1 had one retroperitoneal metastasis collected during second-look surgery 24 months after proctocolectomy, patient no. 2 had retroperitoneal cancer spread diagnosed at the time of primary surgery, from which a sample was collected during autopsy 5 months later, and patient no. 6 had a liver metastasis collected peroperatively.

**Table I** Clinicopathological information on ten patients with longstanding ulcerative colitis (UC)

| *Patient No.* | *Age/Sex* | *Duration of UC (years)* | *Indication for operat.* | *Type of operation* | *Site of main carcinoma* | *Dukes' stage*[a] | *Histological grade*[b] | *Site of metastasis* | *Surv. after prim. op. (mo)* |
|---|---|---|---|---|---|---|---|---|---|
| 1 | 30/M | 13 | Carcinoma | Proctocolectomy | Sigmoid colon | C | Mucinous adenocarcinoma | Peritoneum | 126 |
| 2 | 35/M | 18 | Carcinoma | Right hemicolectomy | Caecum | D | Poorly differentiated | Disseminated[c] | 5[d] |
| 3 | 32/F | 9 | Carcinoma | Proctocolectomy | I[e]: Hepatic flexure | B | Poorly differentiated | None | 127 |
| | | | | | II[e]: Descending colon | A | Well differentiated | | |
| 4 | 36/M | 13 | Carcinoma | Proctocolectomy | Sigmoid colon | A | Moderately differentiated | None | 73 |
| 5 | 34/M | 19 | Carcinoma | Proctocolectomy | Splenic flexure | B | Mucinous adenocarcinoma | None | 26 |
| 6 | 39/M | 12 | Carcinoma | Proctocolectomy | Rectum | D | Poorly differentiated | Liver | 1[d] |
| 7 | 37/M | 20 | Exacerbation | Proctocolectomy | | | | | |
| 8 | 30/F | 8 | Exacerbation and dysplasia[f] | Proctocolectomy | | | | | |
| 9 | 33/F | 23 | Dysplasia | Proctocolectomy | | | | | |
| 10 | 37/M | 21 | Exacerbation | Proctocolectomy | | | | | |

[a]According to the modified Dukes' classification (Dukes, 1932; Turnbull *et al.*, 1967). [b]According to the criteria given by WHO (Morson & Sobin, 1976). [c]Pre- and peroperatively diagnosed metastasis in a lymph node located on the neck, in the retroperitoneal tissue, and in the liver. [d]Until death. [e]This patient had two macroscopical evident carcinomas. [f]In this patient, dysplasia could not be verified in the operative specimen.

**Table II** DNA-indices and degree of dysplasia in tissue samples from ten patients with longstanding ulcerative colitis

| *Tissue sample* | *Patient No.* 1 | 2 | 3 | 4 | 5 | 6 | 7 | 8 | 9 | 10 |
|---|---|---|---|---|---|---|---|---|---|---|
| Macrosc. evident carcinoma | 1.5 (n = 5)[a]<br>1.0[b] (n = 1) | 1.3 (n = 3) | I[c]: 1.0 (n = 1)<br>II[c]: 1.0 (n = 1) | 1.0 (n = 2) | 1.0 (n = 2) | 1.4/2.3[d] (n = 2)<br>2.3 (n = 3) | | | | |
| Flat mucosa | 2.6/high[e] | 1.3/high<br>1.0/high<br>1.0/high | 1.0/high<br>1.0/low<br>1.0/high | 1.7/low<br>1.7/low<br>1.7/ind<br>1.0/no<br>1.7/low<br>1.7/low<br>1.7/ca<br>1.7/low | 1.0/no<br>1.0/high<br>1.7/no<br>1.0/low<br>1.0/low<br>1.0/low<br>1.0/low<br>1.0/low | 2.3/no<br>2.1/no<br>1.0/no<br>1.0/no<br>1.0/no<br>1.0/no<br>1.0/no<br>1.0/no<br>1.0/no<br>1.0/no | 1.0/low<br>1.0/low<br>1.0/low<br>1.0/low<br>1.0/low<br>1.0/low | 1.0/no<br>1.0/no<br>1.0/no<br>1.0/no<br>1.0/no<br>1.0/no | 1.0/high<br>1.0/high<br>1.0/ind<br>1.0/low<br>1.7/no<br>1.7/no | 1.0/low<br>1.0/ind<br>1.0/ind<br>1.0/low<br>1.0/no<br>1.0/ind<br>1.0/no<br>1.0/no<br>1.0/no<br>1.0/no<br>1.0/no |
| Polypoid lesions | 1.4/ca<br>2.1/ca<br>2.0/high | | | | | | | | | |
| Metastasis | 1.5 (n = 2) | 1.3 (n = 1) | | | | 2.1 (n = 1) | | | | |

[a]Number of samples analysed with the same DNA index. [b]One sample from this carcinoma showed only diploid cells. [c]Two macroscopically evident carcinomas were found in this patient. [d]Two samples from this carcinoma showed two aneuploid cell populations, with DNA indices of 1.4 and 2.3, respectively. [e]Denominators indicate grade of dysplasia, high: high grade dysplasia; low: low grade dysplasia; ind: indefinite for dysplasia; no: no dysplasia; ca: infiltrating growth (carcinoma).

### *Histological grading of dysplasia*

The histological grading of epithelial dysplasia was done on hematoxylin-eosin stained sections adjacent to the samples processed for DNA flow cytometry, and performed independently by two pathologists. Whenever discrepancy existed in classification, a joint diagnosis was achieved. The mucosa samples were categorized into: no dysplasia, indefinite for dysplasia, low grade or high grade dysplasia, according to the latest standardised classification of dysplasia in inflammatory bowel disease (Riddell *et al.*, 1983).

### *DNA flow cytometry*

Single cell suspensions were prepared immediately after surgery (in one instance immediately after autopsy). The tissue samples were minced in ice-cold phosphate-buffered saline

(PBS), pH 7.6, followed by nylon mesh filtration (mesh pore size 70 µm) (Seidengazefabrik AG Thal, Switzerland). The cells were fixed and stored in 70% ethanol at 4°C until they were further processed for DNA flow cytometry. The staining for DNA flow cytometry in patients nos. 1, 2 and 3 was done with ethidium bromide (Calbiochem, California, USA) according to the description by Göhde & Dittrich (1971). Prior to the staining, the cells were treated with RNAase (Boehring, Mannheim, Germany), $1 g l^{-1}$ in water, 1 h at 37°C, and pepsin (Orthana, Copenhagen, Denmark), $4 g l^{-1}$ in 0.02 N HCl, 15 min at 37°C. The measurements were performed with an ICP 11 flow cytometer (Phywe AG, Göttingen, Germany). Tissue samples collected from patients nos. four to ten were stained according to the method described by by Crissman & Steinkamp (1982). The cells were incubated with RNAase (Boehring, Mannheim, Germany), $190 \mu g ml^{-1}$ for 30 min in the dark at 20°C, and thereafter stained with the fluorochrome propidium iodide (Sigma Chemical Co., St. Louis, MO, USA), $17 \mu g ml^{-1}$ for 1 h in the dark at 0°C. The analyses were done with an Ortho Cytofluorograph 50H (Ortho Instruments, Westwood, MA, USA).

Mouse spleen lymphocytes were used as an external diploid (2c) DNA control. The cellular amount of DNA was expressed as a DNA index (Hiddemann *et al.*, 1984). A tissue sample was defined as aneuploid when a second detectable population of $G_1$ cells was present with a DNA index $\geqslant 1.10$, otherwise the tissue sample was defined as diploid (or in samples from carcinomas, as near diploid).

### *Statistical analysis*

For differences in distributions, the $\chi^2$ test with Yates correction for small numbers was applied.

### Results

### *DNA ploidy pattern in carcinomas and in tumour-distant flat mucosa*

Six of the ten carcinomas (60%) were aneuploid (Table II, and Figures 1 and 2). The six aneuploid carcinomas were found in patients nos. 1, 2, 4, and 6. These four patients also had aneuploid cell populations in tumour-distant flat mucosa. Three of these four patients (patients nos. 2, 4, and 6) had the same DNA index in tumour-distant flat mucosa as in (one of) the carcinoma(s). Two patients (patients nos. 3 and 5), had near diploid carcinomas. Patient no. 5 had aneuploid cells in tumour-distant flat mucosa, whereas this was not found in patient no. 3. In the three patients with two or more carcinomas (patients nos. 1, 3, and 4) there was no consistent DNA ploidy pattern between the different carcinomas within the same patient (Table II, and Figures 1 and 2).

### *DNA ploidy pattern in flat mucosa from patients without carcinoma*

Twenty-nine mucosa samples were analysed from the four patients without carcinoma. Only two of these samples (from patient no. 9) were aneuploid, both with a DNA index of 1.7 (Table II).

### *Relationship between DNA aneuploidy and dysplasia*

Five of the six carcinoma patients, and one of the four patients without carcinoma had DNA aneuploidy in flat mucosa ($P>0.1$) (Table II). Epithelial dysplasia in flat mucosa was found in five of the six carcinoma patients, and three of the four patients without carcinoma (Table II). High grade dysplasia was demonstrated in four of the six patients with carcinoma, and in one of the four patients without carcinoma.

From the six patients with carcinoma, 32 non-cancerous flat mucosa samples were analysed (Table II). Eighteen samples (56%) showed dysplasia. Seven of the 18 dysplastic mucosa samples (39%) were aneuploid, compared with four of 14 non-dysplastic mucosa samples (29%) (n.s.). Two of the seven mucosa samples with high grade dysplasia (29%), and five of the 11 mucosa samples with low grade dysplasia (45%) were aneuploid, both compared with four of 14 non-dysplastic mucosa samples (both n.s., respectively).

**Figure 1** Flow cytometric DNA histograms of different cell populations, and their corresponding locations in the colorectal specimen from patient 1. This patient also had three polypoid lesions of which the two closest to the main carcinoma are indicated. The 2c peaks indicate the diploid cell population in each tissue sample. **a** One of five similar DNA histograms from the main carcinoma, demonstrating an aneuploid cell population (AN) with DNA index of 1.5. **b** One aneuploid cell population (AN) with DNA index of 1.4, from a cancerous polypoid lesion next to the main carcinoma. **c** One aneuploid cell population (AN) with DNA index of 2.1, from a cancerous polypoid lesion. **d** One aneuploid cell population (AN) with DNA index of 2.6, located in tumour-distant flat mucosa.

From the four non-carcinoma patients, a total of 29 mucosa samples were analysed. Eleven samples (38%) had dysplastic changes, but none of these showed aneuploid cell populations.

Of the total of 61 non-cancerous flat mucosa samples, seven of 29 dysplastic mucosa samples (24%) were aneuploid, compared with six of 32 non-dysplastic mucosa samples (18%) (n.s.). Two of nine mucosa samples with high grade dysplasia (22%), and five of 20 mucosa samples with low grade dysplasia (25%) were aneuploid, both compared with six of the 32 non-dysplastic mucosa samples (both n.s., respectively).

### *DNA ploidy pattern in primary and metastatic carcinoma*

The six patients with carcinomas were followed postoperatively for 26 months or more (Table I). The three patients with metastases all had aneuploid cell populations with the same or similar DNA indices in the primary carcinoma as in the metastatic lesions (Table II, Figure 3).

### Discussion

The colonoscopic surveillance for premalignancy in patients with ulcerative colitis is currently based on screening for dysplastic changes in biopsy samples at repeated examinations (Riddell, 1984). The grading of dysplasia, however, is

**Figure 2** Flow cytometric DNA histograms of different cell populations, and their corresponding locations in patient 4. **a** One of two similar DNA histograms from the main carcinoma, demonstrating a near diploid cell population (2c). **b** The only diploid cell population detected (of a total of eight samples) from tumour-distant flat mucosa. **c** One aneuploid cell population (AN) with DNA index of 1.7, from flat mucosa which histologically showed invasive growth. **d** One of six similar DNA histograms demonstrating one aneuploid cell population (AN) with DNA index of 1.7, from the six indicated locations (■) from tumour-distant flat mucosa.

**Figure 3** Flow cytometric DNA histograms from **a** the main carcinoma, and **b** the retroperitoneal metastasis removed 24 months later from patient 1. **a** Shows one aneuploid cell population from the main carcinoma with DNA index of 1.5. **b** Shows one of two DNA histograms from the metastasis, demonstrating one aneuploid cell population with DNA index of 1.5. In these DNA analysis, both an old sample from the main carcinoma and the freshly removed metastasis were analysed according to the method of Crissman & Steinkamp (1982).

highly subjective (Riddell *et al.*, 1983; Melville *et al.*, 1988; Dixon *et al.*, 1988), and is further complicated if severe inflammation is present (Riddell, 1976; 1983). Among other markers of premalignancy (Rognum *et al.*, 1987*a*) DNA aneuploidy reflecting abnormal chromosome content within cell nuclei, has become the one of greatest interest.

In the present study, aneuploid cell populations could be demonstrated in six of ten colonic carcinomas from patients with ulcerative colitis. We have earlier reported similar proportions of aneuploidy in colorectal carcinomas from larger series of patients without ulcerative colitis (Rognum *et al.*, 1982; 1987*b*; Meling *et al.*, 1991). Other studies have also reported similar proportions of aneuploid carcinomas in patients with ulcerative colitis (Hammarberg *et al.*, 1984; Melville *et al.*, 1988). In one study, however, a much lower proportion of aneuploid carcinomas in ulcerative colitis was found (Fozard *et al.*, 1986), but a small number of samples analysed from each carcinoma might perhaps explain the low rate of aneuploidy detected (Rognum *et al.*, 1980; Fozard *et al.*, 1987; Melville *et al.*, 1987).

In the six patients with carcinoma, high grade dysplasia was present only in four. This indicates that high grade dysplasia is not sufficient as marker for malignant development.

Dysplasia and DNA aneuploidy are different markers of malignant cell transformation. The relationship between them, however, is still not settled. Some studies have demonstrated a significant association between the two markers (Hammarberg *et al.*, 1984; Melville *et al.*, 1988), whereas in other reports, no relationship was found (Fozard *et al.*, 1986; Fischbach *et al.*, 1990). In the present study, we could demonstrate no significant association between DNA ploidy pattern and dysplasia. A part of this discrepancy may be due to the fact that the assessment of dysplasia is highly subjective compared with the more objective evaluation of DNA ploidy pattern (Melville *et al.*, 1988). However, our results suggest that in patients with ulcerative colitis, aneuploidy and dysplasia occur independently in the individual mucosa samples.

A close association has earlier been demonstrated between the presence of carcinoma and aneuploidy in tumour-distant flat mucosa (Hammarberg *et al.*, 1984; Melville *et al.*, 1988). In our study, DNA aneuploidy was found in tumour-distant flat mucosa in five of six patients with carcinoma, and in one of four patients without carcinoma (this patient being the one with high grade dysplasia among the non-carcinoma patients). This indicates that DNA aneuploidy is associated with malignant transformation in the large bowel, but larger series is needed to evaluate the specificity of aneuploidy for cancer development in ulcerative colitis mucosa. However, since approximately one out of three large bowel carcinomas are near diploid, the absence of aneuploidy in flat mucosa does not exclude malignant transformation elsewhere in the mucosa. We could, however, demonstrate that aneuploid cell populations in tumour-distant flat mucosa were not restricted to the patients with aneuploid carcinomas, since two of the three patients with (microscopically evident) near diploid carcinomas, had aneuploid cell populations in tumour-distant flat mucosa (Figure 2).

In aneuploid adenomas, another premalignant lesion in the colonic mucosa, a high percentage of DNA indices between 0.80 and 1.20 has been reported (van den Ingh *et al.*, 1985; Giaretti *et al.*, 1988; Giaretti & Santi, 1990), although DNA indices similar to those of adenocarcinomas have also been reported (Quirke *et al.*, 1986). This low ploidy alteration was suggested to reflect early steps in the aneuploid transformation in the carcinogenesis of colorectal adenomas. In our study, however, the high proportion of aneuploid cell clones with a DNA index of 1.7 (present in nine of 14 aneuploid mucosa samples) (Figure 2) indicates that this ploidy change may be associated with early aneuploid malignancy in ulcerative colitis. The process of carcinogenesis may therefore be different in adenomas and in ulcerative colitis mucosa. This is further supported by a recent study of c-Ki-*ras* mutations, reporting different genetic pathways for tumour progression in colonic mucosa with and without ulcerative colitis (Burmer *et al.*, 1990). A combination of flow cytometric and genetic analyses addressing this question are already in progress in our laboratory.

Five patients had aneuploid carcinomas. Three of them also had aneuploid cell clones in tumour-distant flat mucosa, and these three were the only patients with metastatic disease (Table II). This is, however, interesting, since aneuploid carcinomas are associated with a worse prognosis than diploid ones (Wolley *et al.*, 1982; Armitage *et al.*, 1985; Rognum *et al.*, 1987*b*; 1991). The aneuploid cells in the mucosa from these three patients, all had DNA indices different from 1.7, and in two of the patients, the DNA index was the same as in the main carcinoma. Furthermore, the DNA indices found in the primary carcinomas of these three patients, were also demonstrated in the metastatic carcinomas, respectively (Rognum *et al.*, 1985). The aneuploid cells found in the mucosa of these patients may therefore have reached a point in the carcinogenesis where the ploidy changes are preserved in the cell population that grow invasively, and subsequently establish distant organ metastases.

Within single patients, we frequently observed aneuploid cell populations from separate mucosa samples with similar DNA indices, (Table II, Figure 2) as has also been reported by others (Fozard *et al.*, 1986, Rutegård *et al.*, 1989). We also found the same DNA index in cells from mucosa- and carcinoma samples within single patients. This individual preference of certain ploidy changes might be the result of evenly distributed carcinogenic factors in the colonic mucosa. Furthermore, the genetic constitution of the patient is also likely to influence the ploidy changes (Hsu, 1983). Aneuploid cell clones with identical DNA indices have previously been reported in synchronous carcinomas, supposed to reflect a transluminal metastatic spread from a single lesion (Schwartz *et al.*, 1986). A transluminal spread is, however, highly unlikely in our ulcerative colitis patients, since we found similar DNA indices in separate premalignant lesions (which do not have the potential to metastasise), and also in both premalignant and malignant lesions.

Our results show that aneuploidy occurring in flat mucosa of patients with ulcerative colitis is not confined to dysplastic epithelium. DNA flow cytometric analysis may therefore contribute, together with other markers (Rognum *et al.*, 1987*a*), to the identification of the subset of patients with increased cancer risk, and who might therefore benefit from a closer surveillance. Larger series of patients is however needed to evaluate the specificity of aneuploidy occurring in ulcerative colitis mucosa.

Supported by the Norwegian Cancer Society, A/S Freia Chocolate Factory's Medical Fund, and The Medical Innovation Foundation, Oslo, Norway.

### References

ARMITAGE, N.C., ROBINS, R.A., EVANS, D.F., TURNER, D.R., BALDWIN, R.W. & HARDCASTLE, J.D. (1985). The influence of tumour cell DNA abnormalities on survival in colorectal cancer. *Br. J. Surg.*, **72**, 828.

BARLOGIE, B., RABER, M.N., SCHUMANN, J. & 6 others (1983). Flow cytometry in clinical cancer research. *Cancer Res.*, **43**, 3982.

BURMER, G.C., LEVINE, D.S., KULANDER, B.G., HAGGITT, R.C., RUBIN, C.E. & RABINOVITCH, P.S. (1990). C-Ki-*ras* mutations in chronic ulcerative colitis and sporadic colon carcinoma. *Gastroenterology*, **99**, 416.

CRISSMAN, H.A. & STEINKAMP, J.A. (1982). Rapid, one step staining procedures for analysis of cellular DNA and protein by single and dual laser flow cytometry. *Cytometry*, **3**, 84.

DIXON, M.F., BROWN, L.J.R., GILMOUR, H.M. & 4 others (1988). Observer variation in the assessment of dysplasia in ulcerative colitis. *Histopathology*, **13**, 385.

DUKES, C.E. (1932). The classification of cancer of the rectum. *J. Pathol. Bacteriol.*, **35**, 323.

FISCHBACH, W., MÖSSNER, J., SEYSCHAB, H. & HÖHN, H. (1990). Tissue carcinoembryonic antigen in precancerous and cancerous colorectal lesions. *Cancer*, **65**, 1820.

FOZARD, J.B.J., QUIRKE, P., DIXON, M.F., GILES, G.R. & BIRD, C.C. (1986). DNA aneuploidy in ulcerative colitis. *Gut*, **27**, 1414.

FOZARD, J.B.J., QUIRKE, P. & DIXON, M.F. (1987). DNA aneuploidy in ulcerative colitis. Correspondence. *Gut*, **28**, 642.

GIARETTI, W., SCIALLERO, S., BRUNO, S., GEIDO, E., ASTE, H. & DI VINCI, A. (1988). DNA flow cytometry of endoscopically examined colorectal adenomas and adenocarcinomas. *Cytometry*, **9**, 238.

GIARETTI, W. & SANTI, L. (1990). Tumor progression by DNA flow cytometry in human colorectal cancer. *Int. J. Cancer*, **45**, 597.

GÖDHE, W. & DITTRICH, W. (1971). Impulsfluorometrie – ein neuartiges Durchflussverfahren zur ultraschnellen Mengdenbestimmung von Zellinhaltsstoffen. *Acta Histochem.*, **10** (suppl.), 429.

HAMMARBERG, C., SLEZAK, P. & TRIBUKAIT, B. (1984). Early detection of malignancy in ulcerative colitis. *Cancer*, **53**, 291.

HIDDEMANN, W., SCHUMANN, J., ANDREEFF, M. & 6 others (1984). Convention on nomenclature for DNA cytometry. *Cytometry*, **5**, 445.

HSU, T.C. (1983). Genetic instability in the human population: A working hypothesis. *Hereditas*, **98**, 1.

LENNARD-JONES, J.E., MORSON, B.C., RITCHIE, J.K. & WILLIAMS, C.B. (1983). Cancer surveillance in ulcerative colitis. *Lancet*, **ii**, 149.

LØFBERG, R., TRIBUKAIT, B., ØST, Å., BROSTRØM, O. & REICHARD, H. (1987). Flow cytometric DNA analysis in longstanding ulcerative colitis: a method of prediction of dysplasia and carcinoma development? *Gut*, **28**, 1100.

MELING, G.I., ROGNUM, T.O., CLAUSEN, O.P.F. & 8 others (1991). Association between DNA ploidy pattern and cellular atypia in colorectal carcinomas. A new clinical application of DNA flow cytometry? *Cancer*, **67**, 1642.

MELVILLE, D.M., NORTHOVER, J.M.A., JASS, J.R., SHEPHERD, N.A. & LENNARD-JONES, J.E. (1987). DNA aneuploidy in ulcerative colitis. Correspondence. *Gut*, **28**, 643.

MELVILLE, D.M., JASS, J.R., SHEPHERD, N.A. & 6 others (1988). Dysplasia and deoxyribonucleic acid aneuploidy in the assessment of precancerous changes in chronic ulcerative colitis. *Gastroenterology*, **95**, 668.

MORSON, B.C. & PANG, L. (1987). Rectal biopsy as an aid to cancer control in ulcerative colitis. *Gut*, **8**, 423.

MORSON, B.C. & SOBIN, L.H. (1976). *Histological Typing of Intestinal Tumours*. No. 15. World Health Organization: Geneva.

QUIRKE, P., FOZARD, J.B.J., DIXON, M.F., DYSON, J.E.D., GILES, G.R. & BIRD, C.C. (1986). DNA aneuploidy in colorectal adenomas. *Br. J. Cancer*, **53**, 477.

RANSOHOFF, D.F., RIDDELL, R.H. & LEVIN, B. (1985). Ulcerative colitis and colonic cancer. *Dis. Colon Rectum*, **28**, 383.

RIDDELL, R.H. (1976). The precarcinomatous phase of ulcerative colitis. *Curr. Top. Path.*, **63**, 179.

RIDDELL, R.H. & MORSON, B.C. (1979). Value of sigmoidoscopy and biopsy in detection of carcinoma and premalignant change in ulcerative colitis. *Gut*, **20**, 575.

RIDDELL, R.H., GOLDMAN, H., RANSOHOFF, D.F. & 9 others (1983). Dysplasia in inflammatory bowel disease: standardized classification with provisional clinical applications. *Hum. Pathol.*, **14**, 931.

RIDDELL, R.H. (1984). Dysplasia and cancer in ulcerative colitis: a soluble problem? *Scand. J. Gastroenterol.*, **19** (suppl. 104), 137.

ROGNUM, T.O., THORUD, E., ELGJO, K., BRANDTZAEG, P., ØRJASAETER, H., NYGAARD, K. & CLAUSEN, O.P.F. (1980). DNA flow cytometry (FCM) in carcinomas of the large bowel compared with the two functional cell markers secretory component (SC) and carcinoembryonic antigen (CEA), the histological tumour grade and the clinical stage. A preliminary communication. *Flow cytometry*, **IV**, 417.

ROGNUM, T.O., THORUD, E., ELGJO, K., BRANDTZAEG, P., ØRJASAETER, H. & NYGAARD, K. (1982). Large bowel carcinomas with different ploidy, related to secretory component, IgA and CEA in epithelium and plasma. *Br. J. Cancer*, **45**, 921.

ROGNUM, T.O., THORUD, E. & BRANDTZAEG, P. (1985). Preservation of cytometric DNA distribution and epithelial marker expression after tumor progresson of human large bowel carcinomas. *Cancer*, **56**, 1658.

ROGNUM, T.O., THORUD, E. & LUND, E. (1987*b*). Survival of large bowel carcinoma patients with different DNA ploidy. *Br. J. Cancer*, **56**, 633.

ROGNUM, T.O., BRANDTZAEG, P., ELGJO, K. & FAUSA, O. (1987*a*). Heterogeneous epithelial expression of class II (HLA-DR) determinants and secretory component related to dysplasia in ulcerative colitis. *Br. J. Cancer*, **56**, 419.

ROGNUM, T.O., LUND, E., MELING, G.I. & LANGMARK, F. (1991). Near diploid large bowel carcinomas have better five-year survival than aneuploid ones. *Cancer*, (in press).

ROSENSTOCK, E., FARMER, R.G., PETRAS, R., SIVAK, M.V. Jr., RANKIN, G.B. & SULLIVAN, B.H. (1985). Surveillance for colonic carcinoma in ulcerative colitis. *Gastroenterology*, **89**, 1342.

RUTEGÅRD, J., ÅHSGREN, L., STENLING, R. & ROOS, G. (1989) DNA content and mucosal dysplasia in ulcerative colitis. *Dis. Colon Rectum*, **32**, 1055.

SCHWARTZ, D., BANNER, B.F., ROSEMAN, D.L. & COON, J.S. (1986). Origin of multiple 'primary' colon carcinomas. *Cancer*, **58**, 2082.

TURNBULL, R.B. Jr., KYLE, K., WATSON, F.R. & SPRATT, J. (1967). Cancer of the colon: the influence of the no-touch isolation technic on survival rates. *Ann. Surg.*, **166**, 420.

VAN DEN INGH, H.F., GRIFFIOEN, G. & CORNELISSE, C.J. (1985). Flow cytometric detection of aneuploidy in colorectal adenomas. *Cancer Res.*, **45**, 3392.

WOLLEY, R.C., SCHREIBER, K., KOSS, L.G., KARAS, M. & SHERMAN, A. (1982). DNA distribution in human colon carcinomas and its relationship to clinical behaviour. *JNCI*, **69**, 15.